# Supplementary material for: Transcriptomic Insights into Mechanisms of Early Seed Maturation in the Garden Pea (Pisum sativum L.)
Source: Cells. 2020 Mar 23;9(3):779. doi: 10.3390/cells9030779 (PMC7140803; doi:10.3390/cells9030779)

Sprint−2(10)

Global Summary

%DE = 0.94

genes with fdr < 0.2 = 571 (229 + / 342 −)

genes with fdr < 0.1 = 548 (215 + / 333 −)

genes with fdr < 0.05 = 530 (206 + / 324 −)

genes with fdr < 0.01 = 451 (153 + / 298 −)

genes in genesets = 0

<FC> = 0

<t−score> = −183.72

<p−value> = 0

<fdr> = 0.07

Global Genelist

Rank	ID	log(FC)	p−value	fdr	Metagene	Description
1	TRINITY_DN1	−8.1	2e−14	5e−11	20 x 20	
2	TRINITY_DN4	−0.45	1e−12	9e−10	20 x 4	
3	TRINITY_DN1	−13.3	3e−11	9e−10	20 x 1	
4	TRINITY_DN4	−0.35	5e−11	2e−09	19 x 16	
5	TRINITY_DN3	−0.25	8e−11	2e−09	19 x 10	
6	TRINITY_DN1	−0.05	2e−10	2e−09	12 x 15	
7	TRINITY_DN3	−0.12	2e−10	2e−09	14 x 3	
8	TRINITY_DN5	−0.07	2e−10	5e−09	15 x 11	
9	TRINITY_DN4	−0.06	5e−10	5e−09	14 x 13	
10	TRINITY_DN3	−0.03	5e−10	1e−08	11 x 6	
11	TRINITY_DN6	−1.13	1e−09	1e−08	20 x 1	
12	TRINITY_DN2	−3.15	1e−09	1e−08	20 x 1	
13	TRINITY_DN4	−0.29	1e−09	1e−08	16 x 3	
14	comp42763_c	−0.03	2e−09	1e−08	12 x 7	
15	TRINITY_DN1	−0.11	2e−09	1e−08	14 x 4	
16	TRINITY_DN2	−0.04	2e−09	1e−08	12 x 6	
17	TRINITY_DN2	−0.16	3e−09	1e−08	16 x 6	
18	comp17299_c	−0.02	3e−09	1e−08	10 x 9	
19	TRINITY_DN2	−0.17	3e−09	1e−08	14 x 3	
20	TRINITY_DN6	−0.29	3e−09	2e−08	19 x 7	

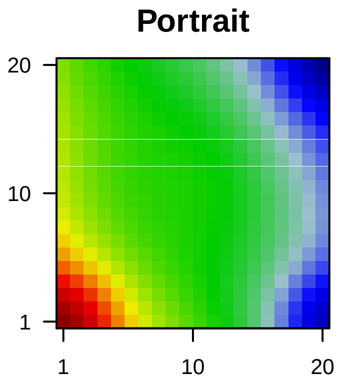

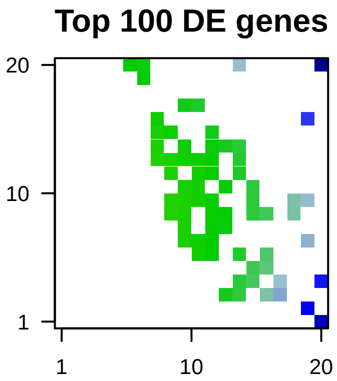

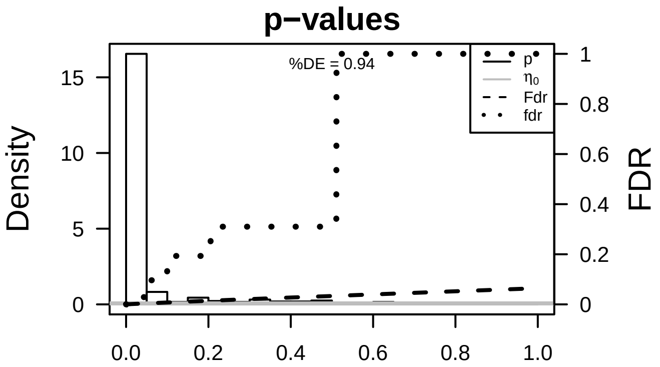

Supplement: Supplementary file 1 [file cells-09-00779-s001.zip › Supplementary materials/FileS3.zip!/FileS2/Hormones - Results/Summary Sheets - Groups/Reports/Sprint.2.10..pdf]
